# Randomized Trial of Probiotics and Nicotinic Acid on Gut Microbiome, Quality of Life in Parkinson’s

**DOI:** 10.1093/geroni/igaf122.2438

**Published:** 2025-12-31

**Authors:** Zaw Wai Htoo, Lydia Zeglin, Linda Yarrow, Mark Haub

**Affiliations:** University of California Cooperative Extension, Novato, California, United States; Kansas State University, Manhattan, Kansas, United States; Kansas State University, Manhattan, Kansas, United States; Kansas State University, Manhattan, Kansas, United States

## Abstract

Evidence suggests that probiotics and nicotinic acid (vitamin B3) may improve symptoms and outcomes of Parkinson’s disease through gut microbiome modulation. This study aimed to determine whether a 12-week placebo-controlled randomized clinical trial would result in changes in constipation, drug efficacy, neuroendocrine levels, and quality of life in people diagnosed with Parkinson’s disease. Forty-eight participants were randomly assigned to one of three groups: (1) probiotics + nicotinic acid, (2) probiotics + placebo, or (3) dual placebo for 12 weeks. Constipation, depression, anxiety, quality of life, mood, symptoms, and nutrition were assessed at baseline, midpoint, and the end of the trial. Blood and stool samples were collected for blood chemistry and gut microbiome analysis using next-generation sequencing (16S rRNA genes, Illumina MiSeq). Statistical significance was set at p < 0.05. The results showed improvements in constipation problems, Movement Disorder Society-Unified Parkinson’s Disease Rating Scale (MDS-UPDRS), Parkinson’s Disease Questionnaire-39 (PDQ-39), quality-of-life scores, and communication issues in probiotics and nicotinic acid groups compared with the placebo group. Blood chemistry remained within normal reference ranges. Supplementation did not change assessments of anxiety, depression, or mood. Gut microbiome analyses showed significant differences in alpha and beta diversity, as well as distinct microbiome compositions related to different interventions, disease status, anxiety, and depression. Several differences became more pronounced after 12 weeks compared with 6 weeks of intervention. Nicotinic acid appeared to have a stabilizing effect on the gut microbiome. The findings suggest that supplementation for twelve weeks may benefit constipation symptoms, gut microbiome, and quality of life measures.

